# The invisible child of personalized medicine

**DOI:** 10.1093/jlb/lsab029

**Published:** 2021-09-06

**Authors:** Katharina Ó Cathaoir

**Affiliations:** Faculty of Law, University of Copenhagen, Karen Blixens Plads 16, 2300 Copenhagen, Denmark

**Keywords:** best interests, genetics, health law, participation, personalized medicine, relational autonomy

## Abstract

This article seeks to bring the invisible child of personalized medicine out from the shadows through legal analysis and empirical data. It uses Denmark as a case to argue that existing policies, laws and practices on personalized medicine neglect the legal and ethical issues specific to children. The article investigates Danish laws and practices in clinical genetics and describes how the law neglects children’s right to self-determination in three ways. Firstly, while child participation is provided for by law, no guidelines have been created to operationalize this norm. Secondly, children’s right not to know is inadequately reflected in current policies. Thirdly, the storage of information from prenatal genetic sequencing raises important issues that are in need of reflection. Several recommendations are made, including for strengthening children’s participation and limiting parents’ access to secondary findings where they relate to untreatable or unpreventable conditions. It furthermore recognizes, however, that children’s self-determination in some circumstances should be viewed relationally due to the interconnected nature of genetics.

## I. INTRODUCTION

Governments, researchers, and other stakeholders increasingly promote personalized medicine as cutting-edge health technology that will improve patient and societal outcomes. Personalized medicine (PM) aims to combine a multitude of data sources, in particular genomic data, but also environmental and lifestyle information, to identify targeted treatment and prevention based on the characteristics of patient groups. Although some have expressed hesitancy as to how revolutionary PM will prove,^1^ several political plans foresee personalized medicine becoming an integral part of publicly funded healthcare in the coming years.^2^

Despite this, as this article will emphasize, existing policies, laws and practices on personalized medicine appear to neglect the legal and ethical issues specific to children. Some examples include the Danish and British strategies,^3^ and the International Consortium for personalized medicine—none of which discuss the legal and ethical issues unique to children.^4^ As this article will discuss, recent amendments to the Danish Health Act relating to personalized medicine did not consider the implications of gathering, storing, and processing children’s genetic data, focusing instead on adult patient autonomy.^5^

This is problematic and deserves scrutiny for several reasons. Firstly, states have obligations to respect and ensure children’s rights under the Convention on the Rights of the Child (CRC).^6^ Secondly, as participation is often framed as a central tenet of personalized medicine,^7^ children’s voices should be included, especially given their classic neglect in health law.^8^ Thirdly, as European and international law gives special protection to genetic data,^9^ the legal and ethical questions raised by, for example, secondary findings related to children’s future health mandate adequate reflection on how best to preserve this ‘special category’ of data.

This article therefore seeks to bring the invisible child of personalized medicine out from the shadows through legal analysis and empirical data. It examines the Danish approach as a case study of how the law governs (or fails to govern) children’s rights and interests within genetics. The article focuses on the Danish legal context: Denmark has established a National Genome Centre (2018), launched a policy strategy on personalized medicine (2016) and amended the Health Act (2018) to facilitate personalized medicine.^10^ Furthermore, the National Genome Centre has selected 12 patient groups to be offered whole genome sequencing as a priority, including children and young persons with cancer and psychiatric conditions, as well as fetal medicine.^11^

While the neglect of the ethical and legal issues specific to children in government strategies and law making does not necessarily mean that children are not being given *any* consideration, it does signal that children’s rights and interests are not center stage. It also suggests that Denmark is not applying long-standing ethical, legal, and social science (ELSI) research on genetics and children to personalized medicine, which this article seeks to remedy.

As self-determination is a central value of the Danish health system and the Health Act, the article explores to what extent the law guarantees the latter value for child genetics patients. In the article, law and practice is presented with reference to international norms on children’s rights, primarily Articles 3 and 12 of the CRC and Council of Europe biomedicine treaties, as well as academic literature and recommendations. Furthermore, ELSI research relating to minors and personalized medicine was reviewed to contextualize and assess the legislation.

The article finds, firstly, that the law seeks to provide a framework for children’s right to information but in line with previous research, practical barriers remain. Secondly, children under 15 do not have legal capacity, which means that they do not control return of secondary findings and thereby, their right not to know is not given legal protection. This is especially clear in prenatal sequencing as, because the fetus lacks legal personhood, secondary findings are not saved in the future child’s health records.

In response, section 4 makes recommendations for how these concerns could be mitigated in a manner that upholds children’s best interests. The article recognizes the complex ethical issues raised by advanced genetic sequencing and acknowledges that self-determination is rarely absolute. It suggests that the law on genetics should be interpreted through the prism of relational autonomy, provided adequate safeguards are ensured. This includes strengthening children’s participation and limiting the longevity of parents’ decisions. Technical solutions should be developed to ensure that data and results are later accessible.

At policy level, the author hopes to contribute to important questions, such as, should children’s genomes be sequenced before they can provide a valid consent, and should advanced sequencing be permitted on healthy children? When is it legitimate for a tax-funded health system to use genetic sequencing as a diagnostic test for children? Which findings should parents and children receive? How are the role and responsibilities of geneticists challenged by personalized medicine?

The article begins by introducing personalized medicine and its implications for children, followed by outlining the methods used in this piece. Thereafter an analysis of Danish law and practice is presented with a focus on children’s self-determination. Finally, recommendations are made for how the concerns explored in section 4 may be addressed.

## II. WHAT IS PERSONALIZED MEDICINE?

Personalized medicine marks a shift from a ‘one size fits all’, population-based approach to healthcare to individualized treatment and prevention. In the twentieth century, as medicine became increasingly evidence-based, randomized clinical trials became the gold standard, with clinical application based on patients’ symptoms or diagnosis.^12^ However, for many patients, the prescribed treatment still does not produce the desired clinical effect or no satisfactory diagnosis is reached. Personalized medicine seeks to address this societal challenge. Big data is central, spanning omics, in particular genetic data, but also patient records and registries. Algorithms analyze these data to identify patterns in datasets and stratify patients, with a view to developing more effective, targeted treatment and prevention.

There are multiple definitions and descriptions of personalized medicine. At a European level, the following definition was relied upon by the Council of the European Union:

[personalized medicine is] a medical model using characteristics of individuals’ phenotypes and genotypes (eg molecular profiling, medical imaging, lifestyle data) for tailoring the right therapeutic strategy for the right person at the right time, and/or to determine the predisposition to disease and/or to deliver timely and targeted prevention.^13^

The Danish strategy emphasizes genetic data as a means of achieving better diagnosis and treatment options, while recognizing molecular profiling, imaging, and user-generated data as increasingly relevant in the coming years.^14^

The benefits of personalized medicine are purported as earlier diagnosis, prevention, and more effective treatments.^15^ Faster diagnosis can spare families the strain of a ‘diagnostic odyssey’ and facilitate access to social supports, as well as inform reproductive decision-making.^16^ While a diagnosis is not a legal requirement for social benefits, Danish parents interviewed in connection with this research project spoke of how a diagnosis was often requested by social welfare authorities and made access easier.^17^ Prospectively, identifying an increased risk of developing certain diseases, notably breast, ovarian, and colon cancers, could allow for increased screening and early intervention.^18^ Pharmacogenetics, including through genome wide association studies identifying biomarkers that signal an adverse reaction to certain drugs, can avoid unsuitable treatments that produce harmful side effects.

Personalized medicine for children is promising for several reasons. There are relatively few clinical trials on children, meaning that childhood diseases remain ill-understood.^19^ Children have instead been treated as ‘small men’,^20^ with off label drug use common. Studies have however shown that some children experience different reactions to certain drugs than adults, notably Warfarin.^21^ In cancer care, personalized medicine is encouraging as childhood cancers carry relatively few mutations (compared to in adults).^22^ Personalized medicine could also have significant implications for rare diseases, which overwhelmingly affect children, and for which clinical trials are often viewed as too expensive.^23^ Several studies point toward the potential of personalized medicine to, for example, treat cystic fibrosis.^24^

Furthermore, while the focus of personalized medicine until now has been on patients with cancer and rare diseases, most strategies, including the current Danish strategies, discuss expanding sequencing to healthy individuals who would act as controls. Several proposals have been made for whole genome sequencing of newborns as part of postnatal screening. For example, in November 2019, the then British Health Secretary announced his ambition for WGS be carried out on all newborns in Britain.^25^ This would allow diseases to be detected in children before they have decision-making capacity and potentially result in their biological material being stored and used as a research ‘resource’. If such strategies were realized, the laws relating to genetics would have relevance for all newborns.

Personalized medicine thereby raises legal and ethical questions, including concerns regarding informed consent, data protection, autonomy, and privacy. The expansion of personalized medicine will require gathering, sequencing, storing, and sharing of genetic and health data. Routine genetic sequencing of children raises distinct concerns, as minors cannot consent. It is therefore timely to analyze how children and genetics are currently regulated.

## III. LOOKING TO CLINICAL GENETICS: DANISH LAW AND PRACTICE

Having introduced personalized medicine as an emerging healthcare technology that (in the Danish Strategy) relies on advanced genetic sequencing, this section outlines current law and practice as a case study on children’s self-determination within clinical genomics. The ensuing analysis relies on a legal doctrinal review of the legislation governing clinical genetics (the Health Act and related secondary legislation and guidance, as well as intersecting legislation governing children in health care). In the below analysis, I evaluate domestic provisions in light of the CRC. The CRC is an internationally binding treaty, which Denmark has ratified but not incorporated into national law.^26^ Therefore, as a dualist legal system, the Convention does not have direct effect in Danish law and a domestic law cannot be set aside based on the CRC alone.^27^ Thus, my criticism of Danish law in relation to the CRC relates to the state’s obligations.

In Denmark, court cases are rare in the field of health law and there have been no judicial pronouncements on how the sections discussed in this article should be or are interpreted in practice. For this reason, there is a gap between the law ‘on the books’ and how the law is applied in the clinic. Those interested in ascertaining the content of the law on children and genetics therefore need to look beyond the legislative text, which this article aims to do.

In order to do so, I conducted semi-structured interviews with 11 clinical geneticists to gain insight into their daily experiences with interpreting and applying the legislation in question. The central focus of each interview was an assessment of whether the law is adequately equipped for the introduction of personalized medicine in Denmark and what barriers, if any, they considered the law posed for their ability to diagnose and treat children.

Clinical geneticists were chosen as they play an important role in interpreting and implementing law on a daily basis.^28^ Previous research has highlighted the ‘significant degree of responsibility’ given to professionals who make key decisions in the local pediatric genetics context.^29^

Clinicians from each of the national clinical genetics departments were interviewed with a view to gaining a nationwide perspective. Danish healthcare is divided into five regions within which there are seven departments of clinical genomics spread throughout Denmark.^30^ Interviewees were selected through contact to the head of the clinic and for their experience with pediatric patients. The interviews were undertaken from September 2018 to May 2019 and therefore reflect the law in force at that time. An interview guide was prepared with a list of interview questions which all interviewees were asked, however, the interviewees were also free to stray if they wanted to raise other issues. The interviews (besides one group interview) were transcribed and subsequently coded with a focus on self-determination.

In the subsequent analysis, I shine a light on the limits to children’s self-determination in the current governance regime. Firstly, despite legislation requiring child participation in medical decision making, practical barriers remain. Secondly, children’s status under law means that their right not to know is not given legal protection, in contrast to the position of adults. Thirdly, the challenge of safeguarding the right to information in prenatal genetic sequencing is introduced. In section 4, the implications of these findings are discussed and suggestions are made for how children’s self-determination could be encouraged through relational autonomy.

### III.A. Children’s Participation in Consent to Genetic Sequencing

In Denmark, the central piece of legislation governing genetics in healthcare is the Health Act (*Sundhedsloven*). The law, *inter alia*, ‘sets out the requirements for the health care system in order to ensure respect for the individual, their integrity, and self-determination’.^31^ The Act regulates a number of important areas, such as the duties of the healthcare system, municipalities and hospitals, patient safety and the rights of patients, including, self-determination, freedom of information, and confidentiality. It entered into force in 2006 and has been amended numerous times since. In July 2018, amendments to the Health Act were adopted to create the national infrastructure deemed necessary to facilitate the introduction of personalized medicine in the clinic, including establishing the National Genome Centre.^32^ The Minister for Health issued secondary legislation in February 2019.^33^

While the Health Act was amended to facilitate personalized medicine, the legal and ethical position of children was not addressed. Thus, as before the amendments, no specialized legislation governs genetic sequencing of children in Denmark.^34^ Instead, general legislation applies, whereby minors can consent to treatment, including genetic analysis from age 15.^35^ In contrast to other countries, ‘mature minors’ do not have capacity to consent to treatment before age 15.^36^ Furthermore, until age 18, the child’s guardian should be informed and consulted in healthcare decisions. This provision reflects a compromise as children aged 15–17 years remain minors, to whom their guardians owe duties of care.^37^ Alternatively, it can be criticized for encroaching on children’s privacy and autonomy, especially in intimate areas such as access to contraception. However, the Agency for Patient Complaints and the Disciplinary Board have found that parents’ entitlement to information can sometimes be limited.^38^ For example, the Disciplinary Board has stated that parents’ access to information should be viewed in light of the treatment’s character, the seriousness of the illness, the type of information, the young person’s age and maturity, as well as the need for follow up at home in light of parents’ caring responsibilities.^39^ Still, more clarity as to the circumstances under which physicians may legitimately withhold information would create greater certainty for all parties.

Furthermore, although minors under 15 do not hold decision-making capacity, the law requires that they be facilitated in participating in healthcare decisions. Healthcare workers must inform the child to the extent they understand the treatment and unless informing would cause harm; the child’s views should be given weight to the extent they are current and relevant.^40^ Ultimately, however, decision-making lies with the guardian, who must make medical determinations based on the child’s interests and needs.^41^ Danish law thereby recognizes elements of article 3 CRC (best interests) and article 12 CRC (participation). However, whereas the CRC requires that all children capable of forming views have a right to express them, the Danish provision states that children should be informed *unless it would cause harm*. The Committee’s interpretation of the Convention, while not binding, does not use harm as a reason for limiting participation (but does specify that states should ensure that the right is not misused in a manner that is harmful to the child).^42^ In fact, the Committee has stated that ‘there is no tension between articles 3 and 12’.^43^ Similarly, General Comment No.4 finds that ‘adolescents need to have a chance to express their views freely and their views should be given due weight’; again without limiting the provision through the prism of harm.^44^ Commentaries on Article 12 likewise do not mention that participation is subject to the harm principle, instead focusing on the benefits of participation to the health of the child.^45^ Specifically, children should be presumed competent, which I consider rendering involvement subject to harm to go against.^46^ It has been underscored that ‘children’s capacity to express views must not be assessed through the experiences and expectations of adults as to what constitutes a valid or appropriate view deserving of recognition or attention’.^47^

In contrast, Caroline Adolphsen concludes that while Danish legislation does not provide as much empowerment as corresponding Norwegian legislation, it complies with the CRC because no age limit is specified for when children should be involved in medical decision-making.^48^ However, in my view, this interpretation is not in keeping with the CRC nor prominent academic literature, given that harm is not mentioned as a criterion for limiting children’s right to be heard.^49^ Focusing on harm risks rendering participation vulnerable to paternalism, instead of recognizing children as rights holders, which is the aim of the CRC.

The prepatory works of the Health Act furthermore suggest that children should normally be informed, stating that children under 15 are often capable of understanding, at least important elements, depending on their development and maturity.^50^ At the same time, the prepatory works also make clear that the parents are expected to attribute weight to their children’s views but the provision does not give physicians the competency to overrule parents’ decisions if they are in conflict with the child’s wishes.^51^

During my interviews with geneticists, it became clear that despite the legislative intent, there are practical barriers to operationalizing participation in genetics.^52^ Central challenges include the complexity of genetics, as well as the uncertainty of potential findings. With advanced sequencing, geneticists no longer determine whether a child suffers from a monogenetic disease but instead try to identify mutations that explain a phenotype, and thereby *could* lead to a diagnosis. A further challenge is that many genetics patients are younger children with cognitive impairments, who often have difficulty concentrating, processing information, and conveying their needs. Finally, for families that do not (fluently) speak Danish, an interpreter is required to ensure that valid consent is given, which can further limit the child’s participation, depending on the quality of interpretation.^53^

Many geneticists, when asked, relayed that they perceived it almost impossible to include children in the consent process. Cassandra, an experienced clinical geneticist with a strong interest in children’s rights, thoughtfully relayed:

In rare cases I do [include children], but many of the children [I see] are mentally disabled, which makes it even more difficult. Besides, these are some complicated issues, so I think that in many cases it would largely be impossible to involve the child. Many times, if they are smaller children, [the parents] do not have them with them because it often just becomes a bit distracting.

This observation aligns with previous research, which suggests that while international and domestic law support child involvement in healthcare decision-making, operationalization remains challenging.^54^ Barriers include protective attitudes of families and physicians, and the sociocultural context.^55^ Yet, interviews with children show that most would like to be informed and allowed to voice preferences,^56^ even where children agree that decision-making be delegated to their parents.^57^ Furthermore, failure to respect children’s right to participation based on lack of resources (such as time) may amount to a state violation of the CRC.^58^

Under the CRC, a child ‘capable of forming his or her own views’ has a right to express such views, which should be ‘given due weight in accordance with the age and maturity of the child’. The formulation of the text suggests that in some circumstances it may not be practicable to involve children in decision-making. However, the CRC Committee considers that Article 12 must be interpreted in light of children’s right to freedom of expression under Article 13 and has taken a more radical approach, stating that all children, regardless of age, should be actively listened to, including through nonverbal forms of communication.^59^ Furthermore, the CRC must be interpreted in light of the Convention on the Rights of Persons with Disabilities (CRPD) and it should not be presumed that children with disabilities are incapable of forming views. Instead, information should be tailored to their level of understanding in light of age and maturity.

While legislation requires children to be informed and their views solicited in decision-making on genetic sequencing, clinicians appear to experience barriers to realizing this obligation. This is particularly problematic for self-determination as the results of advanced genomic sequencing can relate to the child’s future health, as explored in the next section. Furthermore, the CRC Committee has held that providing training on Article 12 to professionals working with children is a core obligation.^60^ Notably, in 2011, the CRC Committee recommended that Denmark ‘ensure, through appropriate training, that all professionals and staff dealing with children’s issues are informed and competent to support the expression of children’s views’.^61^ Yet, child participation is never mentioned in Danish political or legal documents on personalized medicine.

### III.B. The Right Not to Know

Under Danish law, patients have the right to receive adequate healthcare but are generally not entitled to demand specific treatments. Likewise, in genetics, legislation does not specify for which genetic conditions children can be tested and this instead lies within the medical professional’s discretion. In the absence of law, ethical and professional norms have filled the vacuum and traditionally protected the child’s right to know. However, advanced genetic sequencing challenges these (largely) unwritten rules as these techniques can unintentionally reveal secondary findings. This can lead one to question whether children under 15 in fact have a right not to know in genetics.

Geneticists usually refuse to test minors for conditions that debut in adulthood as this is widely regarded as unethical.^62^ The traditional position is that out of respect for children’s autonomy and right to an open future, their opportunity to decide whether to be informed of genetic predispositions should be safeguarded until they reach adulthood; parents should not make this decision. The classic example is Huntington’s disease—an illness that cannot be prevented and will usually manifest in one’s thirties. This position is empirically supported by data that show that adults at risk of Huntington’s disease often decide against testing.^63^

Yet, for some parents, it is difficult to accept this principle. Stephen explains:

I have had parents who became enraged with me because I did not want to examine their 5-year-old child for the mother’s mutation, which would first affect the child as a 50-year-old. … Well, I said, we don’t [screen children]. It is *child abuse* to make presymptomatic diagnostics on minors.

Suspecting the doctor was being slightly ironic in his remarks, I asked again whether he really thought testing is such cases amounts to abuse:

Yes, if the child is [legally] incompetent, there are some who believe that it is child abuse, because the child must be allowed to decide…

Pausing, he stated ‘it has no consequence for their treatment’. The doctor’s experience reflects the pressures that geneticists face from concerned parents, keen to avail of technological advancements. It illustrates geneticists’ gatekeeper role, whereby they must negotiate expectations and sometimes limit access to technology based on unwritten ethical norms. As the doctor highlights, testing for such conditions has no consequences for the child’s current health.

However, personalized medicine challenges this prevailing approach as it relies on next generation sequencing, which carries a risk (1–10 per cent) of secondary findings, ie findings that go beyond the original aim of the testing or the patient’s phenotype.^64^ While analyzing a patient’s genome, variants relating to adult onset diseases may be ‘accidentally’ revealed. To safeguard patients’ right to self-determination, the Danish National Genome Centre has developed a national consent form, which requires patients to decide whether they wish to be informed of findings ‘of importance to health’ (1) where there is an option of treatment or prevention, (2) where there is no treatment or prevention (3) or not to be informed of any secondary findings.^65^

In the case of children aged under 15, their guardians make decisions on secondary findings. On the one hand, this reflects the standard legal position for children. Hens et al. point out that parents already obtain all sorts of information related to their children’s future, such as IQ.^66^ Two geneticists also presented this view; Ingrid stated:

The parents are the guardian of the child and [their decision] is what I have to follow. Just as parents are also responsible if their [child] doesn’t put socks on that day and gets sick or doesn’t put their glasses on and gets run over … I think that is the responsibility of the parents. And if I did it differently, I would be a guardian of the child. I would not think that was right …

Another concurred separately:

The child did not ask to have a genetic test, and of course it is true, but the child also did not ask to come for an eye examination …

The doctor thereby relies on the parent’s legal and moral authority and refuses to substitute her professional judgment of the child’s best interests (even though she would not normally test for this type of mutation). The new technology thereby seems to modify the ethical norm of genetics: while geneticists do not consciously test for adult-onset conditions, they will inform parents if such information is unintentionally revealed as a secondary finding.

This approach can conflict with children’s right not to know, which is fundamental to adult genetics and protected under international and national law.^67^ Under Danish law, patients have the right to information about their health, as well as the right not to receive such information.^68^ In light of patient self-determination, individuals should have the option to refuse health information, such as unforeseen findings or predictions on their prognosis. By informing parents of adult-onset diseases, children’s right not to know is rendered subject to their guardians’ decisions.

Not all geneticists agreed. One geneticist rejected providing parents with information on adult-onset diseases, based on the child’s autonomy. Looking at the consent form, the doctor mused:

I have realized that there are just some things here that don’t work with children. There was, for example, a family with a 14-year-old who was quite cognitively ok … The [parents] said we would like to know all secondary findings. And I said, you know what, you shouldn’t … End of discussion. … You can’t impose things on [children]. I just don’t think that ‘we want to know everything’ should be there when it’s about children.

Here, the geneticist feels strongly that the consent form is unsuitable for children and takes the bold decision to defend their autonomy. She tries to limit parents’ decision making with reference to the child’s best interests, ie that secondary findings revealing diseases that cannot be treated or cured should not be revealed until the child is capable of deciding if she wishes to be informed of this information. This approach regards genetic information as *exceptional*; in contrast, an eye exam, for example, is necessary as it has implications for the child’s current health, while information on genetic predispositions does not have immediate health implications and therefore should not be revealed based on parental consent.^69^

However, even geneticists who strongly defended child autonomy noted that genetic findings *may* have sufficient relevance to others to warrant informing—even if the parents have asked not to be. This is due to the interconnectedness of genetics:

We don’t *want* to tell a child that they carry a BRCA mutation. We would like to wait until they become adults. But it has big implications for the mother or aunt. There we come into conflict between regard for the child and the family. Until now, we have decided to tell them.

The child thereby in a limited way ceases to be the only patient. This echoes the recommendations of the European Society on Human Genetics (ESHG) that if an incidental finding suggests ‘serious health problems’ in either the person being tested or close relatives, where treatment or prevention is possible, the healthcare professional should report the findings.^70^ However, the ESHG also recommends that in the case of children, guidelines should be established to ‘balance the autonomy and interests of the child and the parental rights and needs (not) to receive information that may be in the interest of their (future) family’.^71^

Furthermore, while the law does not draw any red lines in terms of which secondary findings are not in minors’ interests, geneticists appeared to. Several declared with conviction that they would not inform a family if they found a mutation for early onset dementia: ‘*that* I would struggle to put on a child’! This consensus seems to be based on the absence of prevention and treatment options currently available for dementia, coupled with the heavy burden of the information. It suggests that in spite of viewing parents as decision makers, geneticists are generally willing to limit parents’ access to information, where no health intervention is possible. This is in line with doctors’ professional responsibilities to safeguard children’s interests.

Finally, a further unresolved issue raised by Adolphsen is whether parents can receive information about their child aged 15–17 where the child has exercised their right not to know. In genetics, hypothetically this could arise where the child does not want, for example, information on secondary findings but the parent does. Adolphsen suggests that parents should normally be considered to have an independent right of access to information although this could be limited based on the child’s best interests.^72^

The Danish strategies and political process never discuss whether these current practices are in children’s best interests or should be reconsidered. The current consent forms and policies on return of secondary findings appear adult focused, lacking reflection on child patients. This is of concern given that the National Genome Centre has prioritized children with certain diseases.

### III.C. A Right to Information in Prenatal Sequencing?

Finally, an interesting issue that emerged during interviews was the status of the right to information in prenatal genomic sequencing. The latter is offered to couples with a history of fatal fetal abnormality.^73^ A goal of the analysis is to inform the woman’s decision on whether to continue the pregnancy. In Denmark, abortion is a right until 12 weeks gestation.^74^ After this, the woman has to apply to a regional administrative body to be granted permission to have an abortion.^75^ Abortion can be granted, inter alia, based on hereditary disease or disease that will cause *serious* mental or physical illness.^76^

The further question can arise as to whether and which secondary findings geneticists should return in these cases. Previous research suggests that there is no agreement among health professionals as to which findings should be returned.^77^ Interviews conducted with 27 laboratory personnel revealed that they did not routinely report variants of unknown significance (VUS) to clinicians, especially if unconnected to the phenotype.^78^ Uncertainty has been identified as a central element of fetal genome testing due to VUS and secondary findings.^79^

The return of VUS can have unforeseen consequences for the future child, as explained by Irmgard:

… the things I have said [to mothers] about a potential risk continues to be a blemish on the children involved … the mothers are nervous about risks … even if they have given birth to a perfectly healthy child …. how do I keep my information from following the child, even if the child is completely healthy?

In a recent article, Lou, Jensen and Vogel explore mothers’ perspectives on return of VUS in Denmark. While the women’s opinions on receiving uncertain results varied, some struggled with the decision of deciding which results are ‘important’.^80^

Danish law does not help to clarify this uncertainty. The consent form developed by the National Genome Centre uses the term ‘important health related secondary findings’.^81^ While the consent form must be used whenever advanced genetic analysis is performed, it does not have a legal status. As the phrase ‘important’ is not used in the right to information under the Health Act, a conflict could arise if a higher level of information is found to be required by law. This leads to uncertainty for the health professional as to which information they must return and which they can deem too uncertain or remote to share. There is a risk that the Agency for Patient Complaints or the Disciplinary Board will interpret ‘important’ differently to geneticists.

Finally, under Danish law, health professionals are required to record results of procedures in the patient’s health record.^82^ As the fetus lacks legal personhood (and thereby an independent health record) and the mother is the patient, any findings will belong to her health record. If the mother continues the pregnancy, the resulting data may have implications for the future child’s health but not be easily accessible to them. This can have implications for the child’s self-determination, health, and privacy. In contrast, if the woman terminates the pregnancy, the information remains only retrievable by her, although it may also have relevance to the father. Yet, none of these potential concerns are found in the Danish strategies or legislation, despite fetal medicine now forming a central part of the Ministry of Health’s strategy for personalized medicine.

## IV. IMPLICATIONS FOR CHILDREN AND THE FUTURE OF PERSONALIZED MEDICINE

This article has noted several aspects of Danish law on genetics that have neglected children’s rights, focused on the rights to participation and information. These issues should have been addressed or at least discussed in the strategies and political debates on personalized medicine, instead no mention of them is found in publicly available documents.

The first issue relates to children’s legal status; minors under 15 years cannot make decisions relating to their treatment. The strict age limit can be criticized for failing to take children’s evolving capacities and circumstances into account. Research highlights, for example, that children with disabilities and histories of illness may mature earlier and have insights and experiences that their guardians lack.^83^ While legislation seeks to give children a voice in healthcare, operationalizing this norm is challenging. Literature highlights that children should not be assumed incapable of participation; all age groups can struggle to understand the implications of genomics.^84^

It is submitted that guidelines on how to pursue children’s participation in personalized medicine would be beneficial. It is a lost opportunity that the Danish personalized medicine strategies and law reform process have not reflected upon how to address the vulnerabilities of those who lack decision-making capacity. In contrast to the British 100,000 genomes project, the Danish National Genome Centre has not created publicly available, child-centered information. Previous research has identified, for example, training health professionals as an important aspect of improving child participation.^85^ Under Danish law, it is the clinician’s responsibility to determine how a child should be included in a manner that includes their views, while not proving overly burdensome for the child.^86^ Therefore, it is recommended that information directed at children be made available to ensure they are informed of genetic sequencing and the potential results, even when they do not have legal capacity to consent. Furthermore, professionals should be assisted in fulfilling their obligation to inform all patients regardless of legal capacity through for example, training. This would furthermore be in keeping with the recommendations of the CRC Committee, as introduced above.

Secondly, in light of children’s best interests and autonomy, I conclude that the current options for return of secondary findings are unsuitable for children. Instead, it is usually in the best interests of the child that treatable/preventable findings with relevance to the child’s current health be communicated, regardless of parents’ preference. For preventable/treatable findings that relate to the child’s future health, an individual assessment should be carried out as to whether the child is sufficiently mature and early information would thereby be in the child’s best interests. Through participation, children must have a role to play in determining their best interests.^87^

This is in keeping with the interpretations of best interests established by the CRC Committee. As the CRC Committee views it, ‘a larger weight must be attached to what serves the child best.’^88^ For example, Helen Stalford argues that ‘it is generally accepted (in principle at least) that it is right and fair that children’s interests should carry more weight in such decisions because their outcome is likely to have much more profound effects on children in the immediate and longer term’.^89^ The CRC Committee suggests that ‘if a legal provision is open to more than one interpretation, the interpretation which most effectively serves the child’s best interests should be chosen’.^90^ Best interests can also be understood as a form of child autonomy: as a means of ensuring children’s right to an open future, ie ensuring that children enter adulthood with the maximum available options.^91^

However, child autonomy is not absolute, and should in some circumstances be viewed in relation to the family. Maya Sabatello et al. posit that reliance on individualized autonomy may be unsuitable for children and instead favor a relational approach—personalized autonomy.^92^ This approach favors viewing children and guardians’ interests as intertwined, given that child health is highly dependent on the parents. Notably, child-adult dependency may be heightened in a family with a sick child where a parent plays a significant caregiving role.

Still, there should be limits to the level of information that could be provided purely by reference to relational autonomy. To inform parents of a disease that cannot be prevented or treated risks instrumentalizing the child. In her relational account of autonomy and genetics, Anne Donchin reflects:

Children need protection lest they be treated, Cinderella-like, as mere conveniences to advance others’ ends … justice would not be served unless they set constraints on moral sacrifices family members can be expected to make for one another.^93^

Therefore, I recommend that variants related to adult-onset disease with serious implications for close family members could exceptionally be communicated with reference to relational autonomy. Under this view, information regarding, for example, BRCA (increased risk of breast and ovarian cancer) or lynch syndrome (increased risk of colon cancer) could be returned given the risk of a parent developing a life-threatening form of cancer that may require invasive treatment. For preventable or treatable health information that does not need to be acted on immediately, guardians and children should be given the choice to reject receiving such information. Again, depending on the child’s age and maturity, she should play a role in this determination.

In my discussions with geneticists, it was clear that a form of relational autonomy was being practiced. Geneticists reflected on the child’s interpersonal context: did the parents have other children/plan to have other children, were there tensions in the family, reasons in the family history that suggest against informing, such as a history of abuse? The doctors highlighted that the patient—not the family—was their primary concern but were keenly aware that the former could not be completely isolated from the latter.

Paradoxically, geneticists were concerned that by moving genetics into mainstream healthcare, the degree of personalization currently provided would not translate. This is because doctors without specialization in genetics will increasingly order sequencing and return findings to patients. These doctors may not have adequate time to get to know the person behind the patient, nor detailed knowledge of the potential variants that could be returned. While today clinical geneticists with a research background are often involved in interpreting variants, the planned expansion of genetic sequencing would render this unfeasible.

Furthermore, non-treatable/preventable findings should not be communicated in childhood as neither the child’s—nor relational—autonomy provides an adequate justification. While literature suggests that most adults would find this information useful for life planning and personal empowerment, the nature of the information strays far from the phenotype that triggered the sequencing. Furthermore, sharing this information exposes children to privacy risks that are in need to greater consideration, such as ‘sharenting’ on social media or uploading children’s data to nonsecure websites.^94^ Children may also be discriminated against based on genetic diagnoses; in February 2021, the Danish Supreme Court held that an insurance company may ask whether a child had been referred to a clinical genetics department and that the presence of an inherited mutation (without symptoms) amounted to a current condition and was thereby not protected by law.^95^ Thus, revealing that a child carries a BRCA or other mutation could have long-term consequences for the child, such as exclusion from certain insurances.

In the case of prenatal sequencing, the absence of guidelines leaves professionals in a difficult position: do they inform parents of variants of unclear significance based on the patient’s right to information, or withhold data due to their uncertainty and potential to stigmatize a future child? In the Danish context, it could be argued that only information that helps to inform reproductive decision-making, ie indicates serious illness, should be given. If a mother receives VUS that do not relate to serious illness, she is unlikely to be granted an abortion on this basis.^96^

This question is perhaps best resolved with reference to the obligations of the professional, upon which one geneticist reflected:

I work with *sick* children. I am not interested in whether someone is disposed to dementia. That is not realistic as there is neither time nor money for that. Doctors should treat sick patients—not do consumer genetics.

Similarly, Clarke and Thirlaway reflect that, with the expansion of the field, genetics professionals may become ‘lifestyle coaches, requiring a very different set of skills’.^97^ In the Danish, tax-payer funded healthcare system, this would seem an inappropriate use of resources. Similarly, the British 100,000 Genomes project does not transmit secondary findings where the clinician is in doubt of the health consequences of the result or the information is of nonmedical character.^98^ Therefore, while patients have a right to information, it should not be viewed as absolute and should focus on the health of the child.

Finally, the question should be asked as to what becomes of future information revealed about a child or fetus. Given the highly digitized nature of Danish healthcare,^99^ it has been suggested that such information should be stored and made available to the child later in life. For children under 15 this could be achieved, for example, through making such findings inaccessible in the child’s electronic health record until they reach 15 and thereafter informing the child of the possibility of accessing the results. In the case of prenatal sequencing, it has been suggested that a separate fetal health record could be created and should the fetus be born, the information be transferred to the child’s health record.^100^

In conclusion, three years after the entry into force of the legislation establishing the Danish National Genome Centre, uncertainty remains in several areas related to children and prenatal sequencing. These issues should be addressed both in light of children’s rights and to create clarity for clinicians as to their duties.^101^

## V. CONCLUSION

This article has argued that policies and laws on personalized medicine have paid insufficient attention to the legal and ethical issues relating to children. Focusing on Danish legislation and practices in clinical genetics, several children’s rights concerns were mapped. It has also recommended how children’s vulnerabilities could be addressed before personalized medicine is mainstreamed.

Firstly, the consent form for advanced genetic sequencing developed by the National Genome Centre and used nationwide has been found inappropriate for child patients. As children lack legal capacity, their guardians decide which secondary findings should be returned. Although geneticists do not consciously test children for adult onset diseases, if the latter are revealed as secondary findings, the law suggests that they may be communicated to guardians. From a rights perspective, it is positive that the law signals that children should participate in this process, yet there are barriers to its operationalization, which are yet to be addressed and considered in the context of personalized medicine. This has implications for children’s right to self-determination, in particular the right not to know.

Although neither the law nor guidelines developed by the National Genome Centre detail which secondary findings may be returned, there seems to be agreement among the geneticists I spoke with that secondary findings related to diseases like dementia should not be returned to child patients. This is due to an absence of prevention and treatment options, coupled with the heavy burden of the information. It shows that although geneticists generally defer to parents as decision makers, they are willing to limit parents’ access to information where no health intervention is possible.

I recommend that, in light of the best interests’ principle, parents and children should automatically receive secondary findings that can be treated or prevented in childhood. If findings relate to treatable or preventable adult-onset diseases, a concrete relational assessment of the child’s best interests should be undertaken. Parents and children should furthermore be given the opportunity to refuse such findings based on their right not to know. Information that is refused should be stored and made accessible to children once they reach age 15.

Advanced genomic sequencing carries uncertainty, which is heightened in prenatal genomic sequencing. As fetuses lack legal personhood, results are stored in the mother’s health record. It has been suggested that unclear or nonmedical findings should normally not be communicated in light of the aims and function of the Danish healthcare system. If the woman chooses to continue the pregnancy, relevant health information should also be copied to the child’s record to ensure their right to information.

The grand promises of personalized medicine may give rise to unreasonable expectations. Yet, the already pressurized health system should not delve into predictive genomics and instead focus on diagnosing and treating ill children, while pursuing prevention where feasible. Still, when refused by the public system, some parents will instead avail of the growing, lightly regulated and sometimes dubious market of direct to consumer testing.^102^ This renders public discussion and acknowledgment of children’s rights and genetics all the more pressing.

While the concerns discussed in this article currently apply to a limited group of patients, they will take on wider significance should political strategies come to fruition and personalized medicine becomes mainstream healthcare. Regrettably, the preexisting challenges faced by geneticists, families, and children have neither been discussed nor addressed by the legislative process.

## Footnotes

1 Anthony Letai, *Functional precision cancer medicine-moving beyond pure genomics*, 23(9) Nature Medicine 1028 (2017). (Discussing that while genomics in cancer has produced results for some patients, it does not benefit the majority). For the purposes of this paper, I accept that personalized medicine is a political priority, which brings legal and ethical ramifications for patients without assessing whether it will meet its aims.

2 NHS England, Improving Outcomes Through Personalised Medicine (2016); Helsedirektoratet, Nasjonal strategi for persontilpasset medisin i helsetjenesten 2017–2021 (2016); Sundhedsministeriet & Danske Regioner, National strategi for Personlig Medicin 2017–2020 (2016).

3 There is only one mention of children in the NHS England Strategy, p. 10. The French strategy briefly notes that genomic medicine also concerns children (France, Genomic Medicine Plan 2025. (2016). p. 35). An outlier is the Norwegian strategy, which recognizes that patients’ stored genetic data can include information relevant to their children. (Helsedirektoraret, p. 69.) Yet, even this mention sees children as ancillary and fails to recognize that children’s participation in personalized medicine raises complex issues.

4 Notably, Deutsches Zentrum für Luft- und Raumfahrt e. V. (DLR)/DLR Project Management Agency, The ICPerMed Action Plan (2017).

5 See for example, Katharina Ó Cathaoir, *In Search of Solidarity –Personalised Medicine in Denmark*, 21–22 Nordisk socialrättslig tidskrift 65 (2019); Katharina Ó Cathaoir & Mette Hartlev, *Personlig Medicin—en Udfordring for Patientens Rettigheder?*, 101 Juristen 219 (2020).

6 UN General Assembly, Convention on the Rights of the Child, 20 November 1989, United Nations, Treaty Series, vol. 1577, p. 3.

7 Rob Horne, *The Human Dimension: Putting the Person into Personalised Medicine*, 23 New Bioeth 38 (2017). See also, Sundhedsstyrelsen, National strategi for sjældne sygdomme: Statusevaluering og anbefalinger til den fremtidige indsats (2018).

8 Council of Europe, Child-friendly Health Care: the Views and Experiences of Children and Young People in Council of Europe Member States (2011).

9 Regulation (EU) 2016/679 of the European Parliament and of the Council of 27 April 2016 on the protection of natural persons with regard to the processing of personal data and on the free movement of such data, and repealing Directive 95/46/EC (General Data Protection Regulation) (Text with EEA relevance); Council of Europe, Modernised Convention for the Protection of Individuals with Regard to the Processing of Personal Data CM/Inf(2018)15-final.

10 In 2015, the Danish Regions, together with universities and a patient organization, published an Action Plan for Personalized Medicine, with plans to offer genome sequencing to 100,000 Danes (Danske Regioner, Handlingsplan for Personlig Medicin 6 (2015)). In 2016, the Ministry of Health released a National Strategy for Personalised Medicine (Sundheds-og  Ældreministeriet, Personlig Medicin til Gavn for Patienterne: National Strategi for Personlig Medicin 2017–2020, Denmark, 2016).

11 Nationalt Genom Center, *12 patientgrupper valgt til genetisk analyse via ny national infrastruktur* (5 Feburary 2021), https://ngc.dk/nyheder/2021/februar/12-patientgrupper-valgt-til-helgenomsekventering (accessed Apr. 28, 2021).

12 David C. Whitcomb, *What is personalized medicine and what should it replace?*, 9(7) Nature Reviews Gastroenterology & Hepatology 418 (2012).

13 Council conclusions on personalised medicine for patients (2015/C 421/03).

14 Sundhedsministeriet, *supra* note 2, p. 7.

15 Richard Hastings & Abhijit Dixit, *Genomic Medicine for the Paediatrician*, 29(4) Paediatrics and Child Health 185 (2019). See para Diagnostics.

16 Katherine S. Josephs, et al., *Genomics: the power, potential and pitfalls of the new technologies and how they are transforming healthcare*, 19(4) Clin Med (Lond) 269 (2019). See case 2 in particular.

17 Laura E. Navne & Mette N. Svendsen, *De Novo Kin: sharing data, shielding persons, and forging relatedness in precision medicine* (under review).

18 Kyung-Won Hong & Bermseok Oh, *Overview of Personalized Medicine in the Disease Genomic Era*, 43(10) BMB reports 643 (2010).

19 Taha I. Yousif, Khaled Bizanti & Basil Elnazir, *Uses of Personalized Medicine in Current Pediatrics* 5(1) International Journal of Clinical Pediatrics 1 (2016); Maurizio Scarpa, et al., *Personalised Medicine in Paediatrics: Individualising Treatment in Children with Rare Neurological Diseases* 2(2) *EPMA Journal* 231 (2011).

20 Maurizio Scarpa, *Advanced Healthcare for Children and Youth: Application of Targeted Prevention, Predictive Diagnostics and Personalised Treatment Approaches*, 2(2) EPMA J. 137 (2011).

21 Basma Al-Metwali & Hussain Mulla, *Personalised dosing of medicines for children*, 69(5) Journal of Pharmacy and Pharmacology 514 (2017).

22 Barbara C. Worst, et al., *Next-generation Personalised medicine for High-risk Paediatric Cancer Patients—The INFORM Pilot Study*, 65 European Journal of Cancer 91 (2016).

23 Wen Y. Ding, et al., *Big data and stratified medicine: what does it mean for children?*, 104(4) Archives of Disease in Childhood 389 (2019).

24 Diana Bilton, *Personalised medicine in cystic fibrosis must be made affordable*, 15 Paediatric Respiratory Reviews 6 (2014).

25 L. Donnelly, *All children to receive whole genome sequencing at birth, under ambitions laid out by Matt Hancock*, www.telegraph.co.uk/news/2019/11/05/children-receive-whole-genome-sequencing-birth-ambitions-laid, 5 November 2019 (accessed Apr. 28, 2021).

26 Politically, this decision has been explained as based on a desire to maintain political responsibility and not shift power to the courts. See, Fifth Periodic Report Convention on the Rights of the Child Denmark, Social- og Indenrigsudvalget 2015–16 SOU 160 7 (2015).

27 For an analysis of the status of the CRC under Danish law, see, Anne Mørk & Jesper Lindholm, *CRC Rights in Danish Law: Considerations on Methodology*, in Children’s Rights: the Convention on the Rights of the Child in Danish Law 39–41 (Trine Schultz, Anne Mørk and Hanne Hartoft eds., 2020). See also Kirsten Ketscher, *Børns Menneskerettigheder: Om FNs Børnkonvention i Dansk Ret* in Nye Retlige Design (Stine Jørgensen, Helle Krunke, Mette Hartlev & Kirsten Ketscher eds., 2003).

28 In Denmark, clinical geneticists are specialized MDs who have undertaken a four year education in clinical genetics. See, www.dsmg.dk/uddannelser/speciallaege/uddannelse-i-klinisk-genetik/.

29 Gabrielle Bertier, et al., *Unsolved challenges in pediatric whole-exome sequencing: A literature analysis*, 54(2) Critical Reviews in Clinical Laboratory Sciences, 134, 140 (2017).

30 Region Sjælland, *Klinisk Genetisk Afdeling*, www.regionsjaelland.dk/sundhed/geo/roskildesygehus/Afdelinger/Klinisk-Genetisk-Afdeling/om-afdelingen/Sider/genetiske-afdelinger-i-danmark.aspx (accessed Apr. 28, 2021).

31 Bekendtgørelse af sundhedsloven, consolidated law no. 903, Aug. 26, 2019 (hereafter The Health Act), section 1.

32 Folketinget, Forslag til Loven om ændring af sundhedsloven, L 146, 9 February 2018.

33 Sundheds-og Ældreministeriet. BEK no. 360 af 04/04/2019, BEK [executive order] no. 359 April 4, 2019, BEK no. 361 April 4, 2019, BEK no. 355 April 4, 2019.

34 This is in contrast to, for example, Norway, see Lov om humanmedisinsk bruk av bioteknologi m.m. (bioteknologiloven), section 5.7.1.

35 The Health Act, section 17 (1).

36 European Union Agency for Fundamental Rights, Consenting to medical treatment without parental consent (2017).

37 There are several areas where the Health Act allows for parents’ access to information to be limited, eg in relation to guidance on contraception (section 62(2), Health Act), in relation to access to information in the child’s health record (section 37(2), Health Act).

38 Helle Bødker Madsen, Sundhedsret (4th ed., 2018), p. 228.

39 Sundhedsvæsenets Disciplinærnævn (case no. 151603, 26 May 2015).

40 The Health Act, section 20. The same applies to individuals aged over 15 who are judged to not have capacity to consent. The prepatory works make clear that it is the obligation of the healthworker to ensure the child is informed, see, 1997–98, 2. samling—L 15 (som fremsat): Forslag til lov om patienters retsstilling [Bill: Patients’ Rights Act], comments to section 12.

41 Bekendtgørelse af forældreansvarsloven [The Act of Parental Responsibility], Consolidated law no. 776, 7 August 2019, section 2.

42 UN Committee on the Rights of the Child, *General Comment No. 12, The right of the child to be heard* (2009) GE.09- CRC/C/GC/12, 20 July 2009, para 132.

43 Ibid., para 74.

44 CRC *General Comment No. 4: Adolescent Health and Development in the Context of the Convention on the Rights of the Child*, CRC/GC/2003/4, 1 July 2003, para 28

45 Wouter Vandenhole, Gamze Erdem Türkelli & Sara Lembrechts, Children’s Rights: A Commentary on the Convention on the Rights of the Child and Its Protocols (Edward Elgar Publishing, 2019), p. 147; Laura Lundy, Aisling Parkes & John Tobin, *Article 12: The Right to Respect for the Views of the Child*, in The United Nations Convention on the Rights of the Child: A Commentary 398–99 (John Tobin ed., Oxford University Press, 2019).

46 Lundy, Tobin, Parkes, Ibid, p. 400.

47 Ibid, p. 404.

48 Caroline Adolphsen, *Børns Ret til Personlig Fridhed i Velfærdsretten—fra et dansk perspektiv*, in Selvbestemmelse og Tvang i helse-og Omsorgstjenesten 91 (Bjørn Henning Østenstad, Caroline Adolphsen, Eva Naur, Henriette Sinding Aasen, eds., 2018), In contrast, Lundy, Tobin and Parkes state that age limits are not necessarily inconsistent with the CRC, Ibid., p. 414.

49 Adolphsen’s assessment is shared by Mette Hartlev, Ulla Hybel & Peter Bak Mortensen, Sundhed og Jura: Sundhedsretlige perspektiver på sundhedsvæsen, sundhedspersoner og patientrettigheder 175 (Jurist- og Økonomforbundets Forlag, 2nd edition, 2017).

50 1997–98, 2. samling—L 15 (som fremsat): Forslag til lov om patienters retsstilling.

51 Ibid., ‘Children and young people under the age of 15 are often, depending on their development and maturity, able to fully understand the illness, treatment options, etc., or at least essential elements thereof. In that case, their understanding must be included as a significant factor in the guardian’s decision.’ (author’s translation.)

52 For a general discussion of child participation under Danish law, see Hanne Hartoft. *Children’s Right to Participation in Denmark: What Is the Difference between Hearing, Co-Determination and Self-Determination?*, in Children’s Constitutional Rights in the Nordic Countries (Trude Haugli, Anna Nylund, Randi Sigurdsen & Lena R.L. Bendiksen eds., 2019).

53 One study suggests that the quality of Arabic speaking interpreters is low in Denmark, Nada Itani, et al., *Language skills and level of experience among Arabic-speaking healthcare interpreters in Denmark: an explorative study*, 14(6) Divers Equality Health Care 283 (2017).

54 Imelda Coyne & Pamela Gallagher, *Participation in communication and decision-making: children and young people’s experiences in a hospital setting*, 20(15–16) Journal of Clinical Nursing 2334 (2011).

55 Vida Jeremic, et al., *Participation of Children in Medical Decision-Making: Challenges and Potential Solutions*, 13 Journal of Bioethical Inquiry 524 (2016).

56 Mary Donnelly & Ursula Kilkelly, *Participation in Healthcare: the Views and Experiences of Children and Young People*, 19 The International Journal of Children’s Rights (2011); Coyne & Gallagher, Journal of Clinical Nursing, (2011); Lucie Moore & Susan Kirk, *A Literature Review of Children’s and Young People’s Participation in Decisions Relating to Health Care*, 19(15–16) J Clin Nurs. 2215 (2010).

57 Imelda Coyne, et al., *Children’s Participation in Shared Decision-Making: Children, Adolescents, Parents and Healthcare Professionals’ Perspectives and Experiences*, 18(3) European Journal of Oncology Nursing 273 (2014).

58 Laura Lundy, *In Defence of Tokenism? Implementing Children’s Right to Participate in Collective Decision-making*, 25(3) Childhood 340 (2018).

59 CRC Committee, *supra* note 42, para 80.

60 Ibid., para 49.

61 Committee on the Rights of the Child, 7 April 2011, CRC/C/DNK/CO/4.

62 Cara Mand, et al., *Predictive genetic testing in minors for late-onset conditions: a chronological and analytical review of the ethical arguments*, 38(9) Journal of Medical Ethics 519 (2012).

63 Karen E. Anderson, et al., *The choice not to undergo genetic testing for Huntington disease: Results from the PHAROS study*, 96 Clinical Genetics 28 (2019).

64 One Danish hospital reports that secondary findings occur in 9% of advanced sequencing. Sundheds-og-Ældreministeriet, Referat, 2. møde i etisk udvalg for Personlig Medicin (24 November 2017).

65 Nationalt Genom Centre, Informeret Samtykke til omfattende genetisk analyse som led i din behandling (2019).

66 Kristian Hens, et al., *Genetic research on stored tissue samples from minors: a systematic review of the ethical literature*, 149a Am J Med Genet A 2356 (2009).

67 Council of Europe, Convention for the Protection of Human Rights and Dignity of the Human Being with regard to the Application of Biology and Medicine: Convention on Human Rights and Biomedicine Oviedo, 4.IV.1997, ETS No. 164, Article 10; Council of Europe, Additional Protocol to the Convention on Human Rights and Biomedicine concerning Genetic Testing for Health Purposes CETS No. 2013, Article 16; Recommendation CM/Rec (2019) 2 of the Committee of Ministers to member states on the protection of health related data (adopted by the Committee of Ministers on 27 March 2019), appendix to recommendation at 7.6.)

68 Health Act, section 16(1), 2.

69 James S. Ware, et al., *Next generation sequencing for clinical diagnostics and personalised medicine: implications for the next generation cardiologist*, 98(4) Heart 276 (2012).

70 *Whole-genome sequencing in health care: Recommendations of the European Society of Human Genetics* 21 European Journal of Human Genetics 580, 583 (2013).

71 Ibid., 583.

72 Caroline Adolphsen, Mindreåriges Retsstilling i Relation til Behandling (Jurist-og Økonomforbundets Forlag, 2013), p. 69.

73 See further, Sebastian Mohr & Janne Rothmar Herrmann, *The politics of Danish IVF: reproducing the nation by making parents through selective reproductive technologies*, BioSocieties (2021).

74 Health Act, section 43. See further, Janne Rothmar Herrmann Retsbeskyttelsen af fostre og befrugtede æg: om håndteringen af retlige hybrider (Djøf Forlag, 2008).

75 Health Act, section 94.

76 Ibid., section 94(3).

77 S. Narayanan, et al., *Exploring the Issues Surrounding Clinical Exome Sequencing in the Prenatal Setting*, 27(5) J Genet Couns 1228 (2018).

78 Danya F. Vears, et al., *Reporting practices for variants of uncertain significance from next generation sequencing technologies*, 60(10) European Journal of Medical Genetics 553 (2017).

79 Celine Lewis, et al. *Dealing with uncertain results from chromosomal microarray and exome sequencing in the prenatal setting: an international cross-sectional study with healthcare professionals*, Prenatal Diagnosis (2021).

80 Stina Lou, Amalie Hahn Jensen and Ida Vogel, *How are uncertain prenatal genetic results perceived and managed 2 years after they were received? A qualitative interview study*, Journal of Genetic Counseling (2021).

81 Nationalt Genom Center, Informeret samtykke til omfattende genetisk analyse i forbindelse med fosterdiagnostik (1st edition, 2020).

82 Bekendtgørelse om autoriserede sundhedspersoners patientjournaler (journalføring, opbevaring, videregivelse og overdragelse m.v.) no. 530, 28 May 2018.

83 Donna Drew, Ashley Kable & Pamela van der Riet, *The adolescent’s experience of cancer: An integrative literature review*, 26(4) Collegian 492 (2019).

84 Paula M. Saukko, et al., *Patients’ understanding of genetic susceptibility testing in mainstream medicine: qualitative study on thrombophilia*, 12(7) BMC Health Serv Res 82 (2007); Michael J. Dougherty, et al., *The Critical Challenge of Educating the Public About Genetics*, 2(2) Current Genetic Medicine Reports 48 (2014).

85 Donnelly & Kilkelly, *supra* note 56.

86 Caroline Adolphsen, *supra* note 72, p. 103.

87 John Tobin, *Judging the Judges: Are They Adopting the Rights Approach in Matters Involving Children?*, 33 Melbourne University Law Review 579, 591 (2009).

88 Committee on the Rights of the Child, *supra* note 42, para 39.

89 Helen Stalford, *The broader relevance of features of children’s rights law The ‘best interests of the child’ principle*, *in*  Children’s rights in the global human rights landscape: isolation, inspiration, integration? (Eva Brems, et al. eds., 2017).

90 Committee on the Rights of the Child, General comment No. 14 (2013) on the right of the child to have his or her best interests taken as a primary consideration (art. 3, para. 1) United Nations CRC/C/GC/14, 29 May 2013, para. 6(b).

91 Joel Feinberg, *The Child’s Right to an Open Future*, *in*  Whose Child? Children’s Rights, Parental Authority, and State Power (William Aiken & Hugh LaFollette eds., 1980).

92 Maya Sabatello, et al., *Pediatric Participation in Medical Decision Making: Optimized or Personalized?*, 18(3) The American Journal of Bioethics 1 (2018).

93 Anne Donchin, *Autonomy and interdependence: quandaries in genetic decision-making*, *in*  Relational Autonomy: Feminist Perspectives on Autonomy, Agency, and the Social Self 243 (Catriona Mackenzie & Natalie Stoljar eds., 2000).

94 Michael J. S. Beauvais, *Parental Access to Children’s Raw Genomic Data in Canada: Legal Rights and Professional Responsibility,* 12 Frontiers in Genetics 454 (2021).

95 Supreme Court judgment, 26 February 2021, case no. BS-14231/2020-HJR.

96 See further, Hartlev *supra* note 49, p. 533.

97 Angus Clarke & Katie Thirlaway, *Genetic counselling for personalised medicine*, 130 Human genetics 27, 30 (2011).

98 Genomics England, The 100,000 Genomes Project Protocol (2017), p. 44.

99 Klaus L. Høyer, *Data as promise: reconfiguring Danish public health through personalized medicine*, 49(4) Social Studies of Science 531 (2019).

100 This point has been made by Professor Ida Vogel, Aarhus University Hospital.

101 The need for clarity in normative guidelines on sharing of genetic information with family members is discussed in Amica Phillips, et al. *Disclosure of genetic information to family members: a systematic review of normative documents*, Genet Med (2021).

102 See, Santa Slokenberga, European Legal Perspectives on Health-Related Direct-to-Consumer Genetic Testing (Doctoral Thesis, Uppsala University, 2016).

